# Performance of the beta-glucan test for the diagnosis of invasive fusariosis and scedosporiosis: a meta-analysis

**DOI:** 10.1093/mmy/myad061

**Published:** 2023-06-28

**Authors:** Frederic Lamoth, Marcio Nucci, Ana Fernandez-Cruz, Elie Azoulay, Fanny Lanternier, Jens Bremerich, Hermann Einsele, Elizabeth Johnson, Thomas Lehrnbecher, Toine Mercier, Luciana Porto, Paul E Verweij, Lewis White, Johan Maertens, Alexandre Alanio, the 9th European Conference on Infections in Leukemia (ECIL-9), Robina Aerts, Murat Akova, Alexandre Alanio, Diana Averbuch, Ola Blennow, Stéphane Bretagne, Alessandro Busca, Thierry Calandra, Simone Cesaro, Catherine Cordonnier, Rafael De La Camara, Caroline Garcia-Vidal, Lidia Gil, Andreas Groll, Raoul Herbrecht, Hans Hirsch, Peter Hubacek, Giuseppe Indolfi, Csaba Kassa, Katrien Lagrou, Frederic Lamoth, Thomas Lehrnbecher, Per Ljungman, Johan Maertens, Vincent Mallet, Rodrigo Martino, Varun Mehra, Toine Mercier, Malgorzata Mikulska, Marcio Nucci, Livio Pagano, Katia Perruccio, Jose Luis PiÑana, Luciana Porto, Christine Robin, Emmanuel Roilides, Monica Slavin, Jan Styczynski, Frank Tverdek, Paul Verweij, Nadja Hawwa Vissing, Lewis White, Alienor Xhaard, Olga Zajac Spychala

**Affiliations:** Infectious Diseases Service, Department of Medicine, Lausanne University Hospital and University of Lausanne, Lausanne, Switzerland; Institute of Microbiology, Department of Laboratory Medicine and Pathology, Lausanne University Hospital and University of Lausanne, Lausanne, Switzerland; University Hospital, Federal University of Rio de Janeiro, Rio de Janeiro, Brazil; Grupo Oncoclinicas, Brazil; Infectious Disease Unit, Internal Medicine Department, Puerta de Hierro-Majadahonda University Hospital, Fundación de Investigación Puerta de Hierro-Segovia de Arana, Universidad Autónoma de Madrid, Madrid, Spain; Médecine Intensive et Réanimation, APHP, Hôpital Saint-Louis, Paris Cité University, Paris, France; Institut Pasteur, Centre National de Référence Mycoses Invasives et Antifongiques, Groupe de recherche Mycologie Translationnelle, Département de Mycologie, Université Paris Cité, Paris, France; Infectious Diseases Unit, Hopital Necker Enfants malades, APHP, Necker-Pasteur Center for Infectious Diseases and Tropical Medicine, Paris, France; Cardiothoracic Imaging Section, Department of Radiology, Basel University Hospital, 4031 Basel, Switzerland; University Hospital Würzburg, Internal Medicine II, Würzburg, Germany; UK Health Security Agency (UKHSA) Mycology Reference Laboratory, Southmead Hospital, Bristol, UK and MRC Centre for Medical Mycology, Exeter University, Exeter, UK; Division of Pediatric Hematology and Oncology, Hospital for Children and Adolescents, University Hospital, Johann Wolfgang Goethe University, Frankfurt am Main, Germany; Department of Oncology-Hematology, AZ Sint-Maarten, Mechelen, Belgium; Department of Microbiology, Immunology, and Transplantation, KU Leuven, Leuven, Belgium and Department of Hematology, University Hospitals Leuven, Leuven, Belgium; Division of Neuroradiology, Pediatric Neuroradiology Department, University Hospital, Johann Wolfgang Goethe University, Frankfurt am Main, Germany; Department of Medical Microbiology, Radboud University Center, Nijmegen, The Netherlands; Public Health Wales Mycology Reference Laboratory and Cardiff University Centre for Trials Research/Division of Infection and Immunity, UHW, Cardiff, UK; Department of Microbiology, Immunology, and Transplantation, KU Leuven, Leuven, Belgium and Department of Hematology, University Hospitals Leuven, Leuven, Belgium; Institut Pasteur, Centre National de Référence Mycoses Invasives et Antifongiques, Groupe de recherche Mycologie Translationnelle, Département de Mycologie, Université Paris Cité, Paris, France; Parasitology-Mycology laboratory Department, AP-HP, Hôpital Saint-Louis, Paris, France

**Keywords:** Fusarium, Scedosporium apiospermum, Lomentospora prolificans, acute leukemia, transplant recipients, invasive fungal infections, beta-D-glucan

## Abstract

The (1→3)-β-D-glucan (BDG) is a component of the fungal cell wall that can be detected in serum and used as an adjunctive tool for the diagnosis of invasive mold infections (IMI) in patients with hematologic cancer or other immunosuppressive conditions. However, its use is limited by modest sensitivity/specificity, inability to differentiate between fungal pathogens, and lack of detection of mucormycosis. Data about BDG performance for other relevant IMI, such as invasive fusariosis (IF) and invasive scedosporiosis/lomentosporiosis (IS) are scarce.

The objective of this study was to assess the sensitivity of BDG for the diagnosis of IF and IS through systematic literature review and meta-analysis. Immunosuppressed patients diagnosed with proven or probable IF and IS, with interpretable BDG data were eligible. A total of 73 IF and 27 IS cases were included. The sensitivity of BDG for IF and IS diagnosis was 76.7% and 81.5%, respectively. In comparison, the sensitivity of serum galactomannan for IF was 27%. Importantly, BDG positivity preceded the diagnosis by conventional methods (culture or histopathology) in 73% and 94% of IF and IS cases, respectively. Specificity was not assessed because of lacking data. In conclusion, BDG testing may be useful in patients with suspected IF or IS. Combining BDG and galactomannan testing may also help differentiating between the different types of IMI.

## Introduction

Invasive mold infections (IMI) are life-threatening complications in hematologic cancer patients, such as hematopoietic cell transplant recipients or patients receiving intensive chemotherapies for acute leukemia. While invasive aspergillosis and mucormycosis represent the most frequent IMI, invasive fusariosis (IF) and invasive scedosporiosis/lomentosporiosis (IS) account for about 3%–5% of IMI cases in Europe and the United States.^1,2^ This proportion varies geographically; for instance IF is more frequent in Brazil (15%–40% of IMI cases).^3,4^

The diagnosis of IMI is difficult because it often requires invasive procedures such as bronchoscopy or tissue biopsy.^5^ Conventional diagnostic approaches (histopathology and culture) have low sensitivity.^6^ Non-culture tests for the detection of fungal antigens or DNA in serum are useful for the early detection of IMI. Specifically, *Aspergillus*-specific quantitative PCR and/or galactomannan (GM) testing in serum are recommended for the diagnosis of invasive aspergillosis.^5,7^ Quantitative PCR for Mucorales demonstrated appropriate performances for the diagnosis of mucormycosis in serum.^8^ However, diagnostic approaches for IF and IS are limited.^9^ Although blood cultures can detect *Fusarium* and *Scedosporium*/*Lomentospora* spp., their sensitivity is low (10%–40%).^10,11^ Non-culture methods for IF and IS mainly rely on different in-house panfungal PCRs, which are not standardized and not widely available.^9^ Recently, a specific PCR for *Fusarium* spp. detection in serum has been developed with promising results.^12^ Although the GM test displays some cross-reaction with *Fusarium* spp., variable sensitivity results (10%–80%) for the diagnosis of IF in serum have been reported.^12,13^

The (1→3)-β-D-glucan (BDG) is a polysaccharide of the fungal cell wall that can be detected in serum by different commercial kits.^14,15^ BDG testing is approved as a diagnostic tool for the diagnosis of IMI and other invasive fungal infections, such as invasive candidiasis and pneumocystosis.^15,16^ For invasive aspergillosis, an overall sensitivity of 50%–60% has been reported.^15,17^ While BDG testing is not recommended for mucormycosis, it may be useful for the diagnosis of other non-*Aspergillus* IMI, such as IF or IS.^9,15^ However, current data are scarce to assess its actual performance in this setting.

The objective of this study was to assess the sensitivity of BDG testing for the diagnosis of IF and IS in hematologic cancer patients and other immunocompromised populations (e.g., solid organ transplant recipients) by a systematic review of the literature and meta-analysis.

## Methods

### Search strategy and inclusion criteria

A systematic search of the literature was performed in PubMed database (https://pubmed.ncbi.nlm.nih.gov) using the following keywords: ‘beta-glucan’ or ‘glucan’ in combination with ‘Fusarium’, ‘Scedosporium’, ‘Lomentospora’, ‘fusariosis’, ‘scedosporiosis’, and ‘lomentosporiosis’. In addition, a systematic search of all case reports of *Fusarium, Scedosporium*, and *Lomentospora* infections was performed. The papers were excluded on the basis of title/abstract according to the following exclusion criteria: (i) non-clinical studies or non-human infections, (ii) absence of host factors and/or no criteria of invasive fungal infections according to the European Organisation for Research and Treatment of Cancer and Mycoses Study Group Education and Research Consortium (EORTC-MSGERC),^5^ (iii) mixed or concomitant (<4 weeks apart) fungal infections, and (iv) articles in language other than English and for which additional data than those contained in the English abstract were required. Articles containing data about cases fulfilling EORTC–MSGERC criteria of proven or probable IF or IS in immunocompromised patients were screened for the term ‘glucan’. All cases for which individual clinical data were available and for which a result of BDG testing in serum by any validated method was reported were included.

### Data collection

The following data were collected for each individual case: type of underlying disease and immunosuppressive conditions according to EORTC–MSGERC criteria, genus/species of the pathogenic fungus, localization of the fungal disease, presence or absence of fungemia (i.e., positive blood culture for *Fusarium* spp or *Scedosporium*/*Lomentospora* spp.), EORTC–MSGERC classification of the fungal infection, type of BDG test, quantitative value of serum BDG, and result of GM assay if available for IF cases. BDG results were interpreted according to the cut-off of positivity proposed by the manufacturer, which was ≥80 pg/ml for the Fungitell^TM^ assay (Associates of Cape Cod Inc., Falmouth, MA), ≥7 pg/ml for the Wako β-glucan test (FUJIFILM Wako Chemicals, Osaka, Japan) and ≥20 pg/ml for the Fungitec-G assay (Seikagaku, Tokyo, Japan). When the type of BDG assay was not mentioned in the original publication, the cases were included if the BDG result and its interpretation by the authors were not equivocal about the positivity or negativity of the result. Papers reporting only qualitative BDG results (‘positive’ or ‘negative’) were included. In case of multiple testing, the highest BDG value was considered. For IF, results of the GM test (Platelia *Aspergillus* EIA, Bio-Rad Laboratories, Marne-La-Coquette, France) were also collected when available. For studies reporting BDG results and for which individual data could not be collected, authors were contacted and additional data were obtained whenever possible.

### Data analyses

Considering that most included publications were case reports or small case series, a specific tool was designed for the purpose of this meta-analysis in order to assess the quality and completeness of the data. For each included publication, the data were rated as ‘complete’ or ‘partially lacking’ regarding the characteristics of the patients (underlying diseases and immunosuppressive conditions), the characteristics of the fungal infection (EORTC–MSGERC classification, localization of infection), and the BDG data (type of BDG assay, quantitative and qualitative BDG result).

Sensitivity of BDG testing for the diagnosis of proven/probable IF and IS was calculated on the basis of all included cases and for different subgroups (adult vs. pediatric populations, hematologic cancer vs. other underlying conditions, neutropenia vs. no neutropenia, fungemia vs. no fungemia, and localized vs. disseminated infection). For IF, sensitivity of GM was also calculated and compared to that of BDG among cases for which both tests had been performed. For case reports and case-series providing data about the sequence leading to the diagnosis of IF or IS, the timing between the first positive BDG assay and the standard test confirming the diagnosis (culture or histopathology) was assessed.

## Results

### Invasive fusariosis (IF)

**Figure 1.**
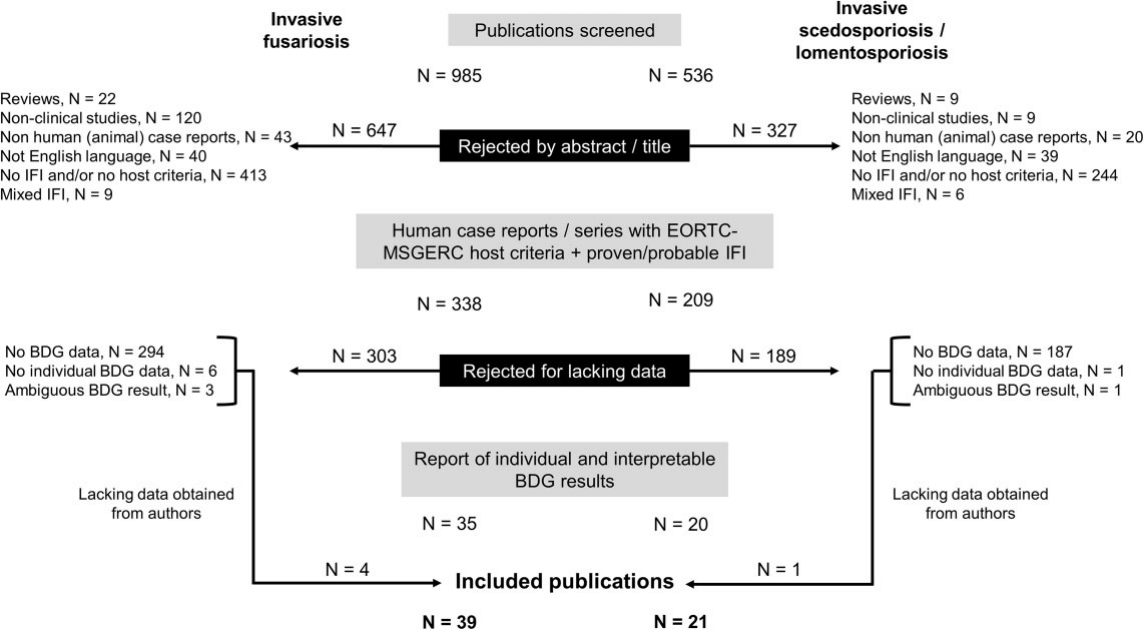
Algorithm for screening and selection of publications. BDG: (1→3)-β-D-glucan, EORTC-MSGERC: European Organisation for Research and Treatment of Cancer and Mycoses Study Group Education and Research Consortium, IFI: invasive fungal infection.

Of 985 publications screened, 39 met the inclusion criteria and were included in the meta-analysis (Fig. 1). Of these, 30 were single case reports and 9 were cohort studies for which individual BDG data were available (Supplementary Figure 1).^12^,^18–55^ Two of these cohorts accounted for a substantial number of cases (*n* = 23, 31.5%).^12,43^ In total, 73 cases of proven or probable IF in patients with EORTC–MSGERC host factors were included. The characteristics of the patients and IF cases are described in Table 1. The majority of patients had hematologic cancers (88%) and were neutropenic (64%). IF was proven in 90% and was associated with disseminated disease in 74% cases and fungemia in 51% cases. Identification of *Fusarium* spp. at species level was available for 48 (66%) cases and *Fusarium solani* complex accounted for 62.5% of them.

**Table 1.** Characteristics of patients and invasive fungal infections.

	Invasive fusariosis (*N* = 73) *N* (%)	Invasive scedosporiosis (*N* = 27) *N* (%)
**Demographic characteristics**		
Female/male/not specified	23 (32)/38 (52)/12 (16)	8 (30)/17 (63)/2 (7)
Adult (≥18 year old)/pediatric/not specified	56 (77)/12 (16)/5 (7)	24 (89)/2 (7)/1 (4)
**Underlying diseases**		
Hematologic cancer	64 (88)	16 (59)
Acute leukemia	31	6
Allogeneic HSCT	12	2
Other hematologic disease	21	8
Solid-organ transplantation	2 (3)	8 (30)
Other or not specified	7 (10)	3 (11)
**Immunosuppressive conditions**		
Neutropenia (yes/no/not specified)	47 (64)/5 (7)/21 (29)	7 (26)/17 (63)/3 (11)
Immunosuppressive therapy (yes/no/not specified)^1^	21 (29)/34 (47)/18 (25)	21 (78)/5 (19)/1 (4)
**EORTC–MSGERC classification**		
Proven/probable	66 (90)/7 (10)	23 (85)/4 (15)
**Site of invasive fungal infection**		
Single site/disseminated (>1 site)/not specified	14 (19)/54 (74)/5 (7)	15 (56)/12 (44)/0 (0)
Fungemia (yes/no/not specified)	37 (51)/32 (44)/4 (5)	8 (30)/19 (70)

EORTC–MSGERC: European Organisation for Research and Treatment of Cancer and Mycoses Study Group Education and Research Consortium, HSCT: hematopoietic stem cell transplantation

^1^ Including mainly corticosteroids and calcineurin inhibitors.

BDG was detected by the Fungitell assay in 53 (73%) cases and by the Wako or Fungitec-G assays in 5 (7%) cases and the type of test was not specified in the remaining 15 cases (21%). The BDG test was interpreted as positive in 56/73 cases leading to a sensitivity of 76.7% (Table 2). Subanalyses in different subgroups did not show significant differences, although sensitivity tended to be higher in patients with disseminated IF and fungemia, and to be lower in the pediatric population (Table 2). No significant difference was observed between infections due *to F. solani* complex vs. other *Fusarium* spp. (73.3% vs. 88.9%, *P* = 0.3).

**Table 2.** Sensitivity of BDG for the diagnosis of IF and IS.

	Overall	Adult Pediatric	Hematologic cancer Other conditions	Neutropenia No neutropenia	Fungemia No fungemia	Single site Disseminated
**IF**	56/73 (76.7)	46/56 (82.1)	48/64 (75.0)	37/47 (78.7)	30/37 (81.1)	10/14 (71.4)
		8/12 (66.7)	5/6 (83.3)	4/5 (80.0)	23/32 (71.9)	43/54 (79.6)
**IS**	22/27 (81.5)	20/24 (83.3)	12/16 (75.0)	6/7 (85.7)	6/8 (75.0)	13/15 (86.7)
		1/2 (50.0)	10/11 (90.9)	14/17 (82.4)	16/19 (84.2)	9/12 (75.0)

Number of cases with positive BDG/total number of cases (sensitivity in percentage)

Concomitant results of GM testing were reported in 44 cases and were positive in 12 of them (sensitivity 27.3% compared to 71.0% for BDG).

The timing of BDG positivity compared to the diagnosis of IF obtained by histopathology or culture was assessed for 33 cases. BDG positivity preceded the positive culture/histopathology in 24 (73%) cases and was concomitant to it in 3 (9%) cases, while in 6 (18%) cases the first positive BDG result was obtained after IF diagnosis.

### Invasive scedosporiosis/lomentosporiosis (IS)

Of 536 publications screened, 21 met the inclusion criteria and were included in the meta-analysis (Fig. 1). Of these, 18 were single case reports and 3 were cohort studies providing individual BDG data for a small number of cases (1–4 patients) (Supplementary Figure 1).^18,23^,^56–74^ In total, 27 cases of proven or probable IS (16 scedosporiosis, 10 lomentosporiosis, and 1 mixed infection) in patients with EORTC–MSGERC host factors were included. The characteristics of the patients and IS cases are described in Table 1. Most patients had hematologic cancers (59%) or solid-organ transplantation (30%). Neutropenia was reported in 26% of them, while 78% received immunosuppressive therapies. IS was proven in 85% cases with disseminated disease and fungemia in 44% and 30% cases, respectively. BDG was measured by the Fungitell assay in 13 (48%) cases, by the Wako or Fungitec-G assays in 7 (26%) cases, while the type of test was not specified in 7 cases (26%). The BDG test was interpreted as positive in 22/27 cases leading to a sensitivity of 81.5% (Table 2). Sub-analyses in different subgroups did not show significant differences (Table 2). Results were similar between infections due *Lomentospora prolificans* vs. *Scedosporium apiospermum* complex (80.0% and 81.2% sensitivity, respectively).

The timing of BDG results could be assessed in 16 cases. BDG positivity was found to precede IS diagnosis by culture/histopathology in 15 (94%) cases and to be concomitant to it in one case.

## Discussion

The use of BDG testing for the diagnosis of IMI is limited globally by its modest sensitivity (50%–70%) and limited specificity (80%–90%).^15,75,76^ A meta-analysis restricted to cohort studies of hematologic cancer patients showed a 50%–60% sensitivity of the BDG test in this setting.^17^ Moreover, a positive BDG cannot make the distinction between the different fungal pathogens. For these reasons, there is currently a weak recommendation to support the use of BDG testing for IMI diagnosis among patients with hematologic cancer or other immunosuppressive conditions and BDG has been removed from the mycological criteria of probable IMI in the updated revised EORTC–MSGERC definitions.^5,7,15,77^ More sensitive and more specific fungal biomarkers in serum, such as the GM test for invasive aspergillosis and specific quantitative PCRs for *Aspergillus* or Mucorales are usually recommended.^5,7,8,77,78^ However, there is currently no specific or validated assay for the diagnosis of IF or IS, although the development of specific PCR looks to be a promising approach.^12^

While the BDG test is not specific for these fungal diseases, our meta-analysis suggests a relatively good sensitivity for the detection of IF (77%) and IS (82%), which is actually higher than that reported for IA (50%–60%).^17^ Considering a relatively low incidence (i.e., 1%–2%) of these IMI in high-risk populations, this would correspond to a negative predictive value >99.5%.

IF and IS are extremely severe IMI that should be promptly treated, thus requiring rapid diagnosis. While blood cultures can detect *Fusarium, Lomentospora*, and *Scedosporium* spp., their yield is limited (40%, 45%, and 6%, respectively) and the time to diagnosis is relatively long (2–3 days).^11,79^ It is important to note that BDG sensitivity remained high in our sub-analysis limited to non-fungemic cases (Table 2). Moreover, we showed that a positive BDG test preceded diagnosis by conventional methods (culture or histopathology) in a majority of cases. Therefore, BDG testing may represent an interesting diagnostic tool for the early detection of IF and IS, as prompt recognition and management of these severe diseases may improve their prognosis.

While the cross-reactivity of GM with *Fusarium* spp. is well described,^13,38^ our results indicate that GM sensitivity in serum for IF diagnosis was only 27%. Of note, these data were mainly derived from two case-series (Nucci et al. and Dellière et al.) displaying very distinct results with GM sensitivity of 77% (10/13 cases) and 0% (0/10 cases), respectively.^12,43^ Such discrepancies might be related to the type of *Fusarium* spp. with better GM sensitivity in studies where *F. solani* predominates and lower sensitivity when there is a majority of other *Fusarium* spp.^12,13,43^ Of note, we did not observe a lower sensitivity of BDG among the non-*solani Fusarium* spp. in the present meta-analysis although their number was limited.

Although IF and IS represent the most frequent IMI after invasive aspergillosis and mucormycosis, other pathogenic molds (e.g., other hyalohyphomycetes, dematiaceous fungi) may be considered in case of a positive BDG result.^23^ We could not assess the sensitivity of BDG for the detection of these rare molds because of the paucity of data. Some data suggest that BDG testing may also be useful for the diagnosis of endemic mycoses, such as histoplasmosis.^80^

Some limitations of the present study should be outlined. First, the majority of the included publications consisted of single case reports, which may not be representative of the usual epidemiology and clinical presentation of these diseases. Inclusion of more severe and mostly proven IF or IS cases may have overestimated the sensitivity of BDG in this setting, but limits the uncertainty related to probable cases. Second, for IF, two cohort studies accounted for about one-third of cases (23/72),^12,43^ which may also represent a bias. Third, the quality of the selected publications is hampered by the use of diverse BDG kits with some lack of precision about the type of test or the quantitative results in some of them. Fourth, results of sub-analyses in different subgroups of patient should be interpreted cautiously (e.g., pediatric population) because of the small number of cases. Fifth, while ongoing antifungal prophylaxis may alter BDG sensitivity,^46^ its impact could not be assessed in the present study because these data were lacking in about half of cases and only few of the remaining cases had received anti-mold prophylaxis. Sixth, while we could determine that BDG positivity preceded the diagnosis of proven IF or IS by culture or histopathology in a majority of cases, the information about the exact difference (i.e., number of days between first positive BDG result and culture/histopathology) was available for few of them. Moreover, these results may be biased by the fact that BDG was performed upon clinical suspicion and not as a screening strategy in most cases. Finally, the available dataset did not allow us to draw any conclusion about the specificity of BDG in this setting. Only one study reported data of BDG specificity for IF diagnosis using a small number (*N* = 13) of matched controls (same underlying conditions and no criteria of invasive fungal disease).^43^ In this study, the specificity was 54%, which would result in a positive predictive value of only 5% considering the incidence of IF in this center. Important variations of BDG specificity have been reported across studies, which may be related to the type of patients’ underlying conditions, the use of antifungal prophylaxis, and also possibly the handling of samples in the laboratory.^15,81^ A meta-analysis restricted to cohorts of adult hematologic cancer patients suggested an overall BDG specificity of about 90%, which could be increased to 99% with the requirement of two consecutive positive tests.^17^ However, specificity of BDG was shown to be lower (50%–70%) in non-hematologic cancer patients (e.g., solid-organ transplant recipients) or in children/neonates.^82,83^

In conclusion, this systematic review and meta-analysis provides the largest dataset about the sensitivity of BDG testing in serum for the diagnosis of IF and IS. The good sensitivity suggests a high negative predictive value for these rare mold infections, although this should be interpreted cautiously in the local epidemiological context and according to the level of clinical suspicion (pre-test probability). There is more concern about the specificity and positive predictive value, in particular, when the test is used in non-hematologic patients or as a screening test in high-risk hematologic patients. Therefore, BDG testing might be considered as an adjunctive diagnostic tool to rule out IF/IS in patients with low clinical suspicion or to guide antifungal therapy in patients with moderate/high clinical suspicion. Combining GM and BDG may also be useful for the distinction between different types of IMI (i.e., invasive aspergillosis, mucormycosis or other IMI such as IF or IS).

## Supplementary Material

myad061_Supplemental_File

## Appendix

**List of collaborators (participants to ECIL-9)**

Robina AERTS, Murat AKOVA, Alexandre ALANIO, Diana AVERBUCH, Ola BLENNOW, Stéphane BRETAGNE, Alessandro BUSCA, Thierry CALANDRA, Simone CESARO, Catherine CORDONNIER, Rafael DE LA CAMARA, Caroline GARCIA-VIDAL, Lidia GIL, Andreas GROLL, Raoul HERBRECHT, Hans HIRSCH, Peter HUBACEK, Giuseppe INDOLFI, Csaba KASSA, Katrien LAGROU, Frederic LAMOTH, Thomas LEHRNBECHER, Per LJUNGMAN, Johan MAERTENS, Vincent MALLET, Rodrigo MARTINO, Varun MEHRA, Toine MERCIER, Malgorzata MIKULSKA, Marcio NUCCI, Livio PAGANO, Katia PERRUCCIO, Jose Luis PIÑANA, Luciana PORTO, Christine ROBIN, Emmanuel ROILIDES, Monica SLAVIN, Jan STYCZYNSKI, Frank TVERDEK, Paul VERWEIJ, Nadja Hawwa VISSING, Lewis WHITE, Alienor XHAARD, Olga ZAJAC SPYCHALA
