# Supplementary material for: Performance of the beta-glucan test for the diagnosis of invasive fusariosis and scedosporiosis: a meta-analysis
Source: Med Mycol. 2023 Jun 28;61(7):myad061. doi: 10.1093/mmy/myad061 (PMC10405209; doi:10.1093/mmy/myad061)
Supplement: myad061_Supplemental_File [file myad061_supplemental_file.docx]

**Supplementary Table 1. Publications included in the meta-analysis for invasive fusariosis**

| **First author, year [reference]** | **Study type** | **N cases** | **Quality / completeness of data** | | |
| --- | --- | --- | --- | --- | --- |
|  |  |  | **Patients** | **Fungal infections** | **BDG test and result** |
| Yoshida, 1997 [1] | Case-series | 1 |  |  |  |
| Okada, 2000 [2] | Case-report | 1 |  |  | c |
| Miyazaki, 2001 [3] | Case-report | 1 |  |  | c |
| Nishio, 2002 [4] | Case-report | 1 |  |  | c, d |
| Odabasi, 2004 [5] | Cohort | 2 |  |  |  |
| Ostrosky-Zeichner, 2005 [6] | Cohort | 3 | a | b | d |
| Gupta, 2007 [7] | Case-report | 1 |  |  | c, d |
| Cuetara, 2009 [8] | Case-series | 1 |  |  |  |
| Patterson, 2009 [9] | Case-report | 1 |  |  | c, d |
| Bellanger, 2011 [10] | Cohort | 3 | a |  |  |
| Kawashima, 2012 [11] | Case-report | 1 |  |  |  |
| Mikulska, 2012 [12] | Case-report | 1 |  |  | c |
| Inano, 2013 [13] | Case-report | 1 |  |  | c |
| Nakai, 2014 [14] | Case-report | 1 |  |  | c |
| Rose, 2014 [15] | Cohort | 2 |  | b |  |
| Garcia-Ruiz, 2015 [16] | Case-series | 1 | a |  |  |
| Hu, 2015 [17] | Case-report | 1 |  |  |  |
| Koltze, 2015 [18] | Cohort | 1 | a |  |  |
| Ramos-Garcia, 2015 [19] | Case-report | 1 |  |  |  |
| Ricna, 2015 [20] | Case-report | 1 |  |  |  |
| Angebault, 2016 [21] | Cohort | 4 | a |  |  |
| Azoulay, 2016 [22] | Cohort | 2 |  |  |  |
| Sheela, 2017 [23] | Case-report | 1 |  |  |  |
| Uemura, 2018 [24] | Case-report | 1 |  |  |  |
| Yoshida, 2018 [25] | Case-report | 1 |  |  | c, d |
| Dehal, 2019 [26] | Case-report | 1 |  |  |  |
| Konuma, 2019 [27] | Case-report | 1 |  |  |  |
| Nucci, 2019 [28] | Cohort | 13 |  |  |  |
| Szvalb, 2019 [29] | Case-report | 1 |  |  | c |
| Bidecci, 2020 [30] | Case-series | 1 |  |  |  |
| Fernandez-Cruz, 2020 [31] | Cohort | 4 | a |  |  |
| Fujishita, 2020 [32] | Case-report | 1 |  |  | c |
| Hoshino, 2020 [33] | Case-report | 1 |  |  |  |
| Ning, 2021 [34] | Case-report | 1 |  |  | c, d |
| Brent, 2022 [35] | Case-report | 1 |  |  | c |
| Dellière, 2022 [36] | Cohort | 10 | a |  |  |
| Jabr, 2022 [37] | Case-report | 1 |  |  | c, d |
| Risum, 2022 [38] | Case-report | 1 |  |  | c |
| Kao, 2023 [39] | Case-report | 1 |  |  |  |

The grey color indicates fields where data are partially lacking.

a: lacking data about underlying conditions (disease or type of immunosuppression)

b: lacking data about localization of invasive fungal infections

c: type of BDG assay not specified

d: quantitative value of BDG result not specified (qualitative result only: positive or negative)

**Supplementary Table 2. Publications included in the meta-analysis for invasive scedosporiosis and lomentosporiosis**

| **First author, year [reference]** | **Study type** | **N cases** | **Quality / completeness of data** | | |
| --- | --- | --- | --- | --- | --- |
|  |  |  | **Patients** | **Fungal infections** | **BDG test and result** |
| Ochiai, 2003 [40] | Case-report | 1 |  |  | c, d |
| Cuetara, 2009 [8] | Case-series | 2 |  |  |  |
| Ohashi, 2011 [41] | Case-report | 1 |  |  |  |
| Takeuchi, 2011 [42] | Case-report | 1 |  |  | c |
| Yu, 2013 [43] | Case-report | 1 |  |  |  |
| Nishimori, 2014 [44] | Case-report | 1 |  |  |  |
| Uno, 2014 [45] | Case-report | 1 |  |  | c |
| Inoda, 2015 [46] | Case-report | 1 |  |  | c, d |
| Ochi, 2015 [47] | Case-report | 1 |  |  |  |
| Angebault, 2016 [21] | Cohort | 3 | a |  |  |
| Tamaki, 2016 [48] | Case-report | 1 |  |  | c |
| Levesque, 2017 [49] | Cohort | 1 |  |  |  |
| Kondo, 2018 [50] | Case-report | 1 |  |  |  |
| Mitomo, 2018 [51] | Case-report | 1 |  |  | c |
| Sakata, 2018 [52] | Case-report | 1 |  |  |  |
| Bronnimann, 2021 [53] | Cohort | 4 |  |  |  |
| Suzuki, 2021 [54] | Case-report | 1 |  |  |  |
| Bourlond, 2022 [55] | Case-report | 1 |  |  |  |
| Gao, 2022 [56] | Case-report | 1 |  |  | c |
| Garnham, 2022 [57] | Cohort | 1 | a |  | d |
| Saini, 2022 [58] | Case-report | 1 |  |  |  |

The grey color indicates fields where data are partially lacking.

a: lacking data about underlying conditions (disease or type of immunosuppression)

b: lacking data about localization of invasive fungal infections

c: type of BDG assay not specified

d: quantitative value of BDG result not specified (qualitative result only: positive or negative)

**REFERENCES**

1. Yoshida M, Obayashi T, Iwama A, Ito M, Tsunoda S, Suzuki T, Muroi K, Ohta M, Sakamoto S, Miura Y. 1997. Detection of plasma (1 --> 3)-beta-D-glucan in patients with Fusarium, Trichosporon, Saccharomyces and Acremonium fungaemias. J Med Vet Mycol 35:371-4.

2. Okada H, Hamatani S, Kondo M, Imai T, Itoh S, Isobe K, Onishi S. 2000. Successful treatment of disseminated Fusarium infection in an infant with leukemia. Int J Hematol 72:494-8.

3. Miyazaki M, Miyakoshi S, Kami M, Mori M, Kishi Y, Inagawa H, Machida U, Matsumura T, Kawagoe S, Ueyama J, Morinaga S, Matsushita H, Muto Y. 2001. Systemic fusariosis after a preparative regimen including thiotepa, VP-16 and busulfan used for blood stem cell transplantation in Hodgkin's disease. Leuk Lymphoma 40:441-4. <https://doi.org/10.3109/10428190109057947>

4. Nishio H, Sakakibara-Kawamura K, Suzuki T, Utsumi T, Kinoshita S. 2002. [An autopsy case of Ph1--positive acute lymphoblastic leukemia with disseminated infection of Fusarium solani]. Kansenshogaku Zasshi 76:67-71. <https://doi.org/10.11150/kansenshogakuzasshi1970.76.67>

5. Odabasi Z, Mattiuzzi G, Estey E, Kantarjian H, Saeki F, Ridge RJ, Ketchum PA, Finkelman MA, Rex JH, Ostrosky-Zeichner L. 2004. Beta-D-glucan as a diagnostic adjunct for invasive fungal infections: validation, cutoff development, and performance in patients with acute myelogenous leukemia and myelodysplastic syndrome. Clin Infect Dis 39:199-205. <https://doi.org/10.1086/421944>

6. Ostrosky-Zeichner L, Alexander BD, Kett DH, Vazquez J, Pappas PG, Saeki F, Ketchum PA, Wingard J, Schiff R, Tamura H, Finkelman MA, Rex JH. 2005. Multicenter clinical evaluation of the (1-->3) beta-D-glucan assay as an aid to diagnosis of fungal infections in humans. Clin Infect Dis 41:654-9. <https://doi.org/10.1086/432470>

7. Gupta S, Almyroudis NG, Battiwalla M, Bambach BJ, McCarthy PL, Proefrock AD, Ball D, Paplham P, Varma A, Kwon-Chung J, Segal BH. 2007. Successful treatment of disseminated fusariosis with posaconazole during neutropenia and subsequent allogeneic hematopoietic stem cell transplantation. Transpl Infect Dis 9:156-60. <https://doi.org/10.1111/j.1399-3062.2006.00189.x>

8. Cuetara MS, Alhambra A, Moragues MD, Gonzalez-Elorza E, Ponton J, del Palacio A. 2009. Detection of (1-->3)-beta-D-glucan as an adjunct to diagnosis in a mixed population with uncommon proven invasive fungal diseases or with an unusual clinical presentation. Clin Vaccine Immunol 16:423-6. <https://doi.org/10.1128/CVI.00009-09>

9. Patterson TF, Mackool BT, Gilman MD, Piris A. 2009. Case records of the Massachusetts General Hospital. Case 22-2009. A 59-year-old man with skin and pulmonary lesions after chemotherapy for leukemia [corrected]. N Engl J Med 361:287-96. <https://doi.org/10.1056/NEJMcpc0809065>

10. Bellanger AP, Grenouillet F, Henon T, Skana F, Legrand F, Deconinck E, Millon L. 2011. Retrospective assessment of beta-D-(1,3)-glucan for presumptive diagnosis of fungal infections. APMIS 119:280-6. <https://doi.org/10.1111/j.1600-0463.2011.02728.x>

11. Kawashima N, Yoshida N, Matsushita N, Ito M, Matsumoto K, Kato K. 2012. Intra-articular injection of voriconazole for Fusarium solani arthritis after bone marrow transplantation. J Infect 65:366-7. <https://doi.org/10.1016/j.jinf.2012.06.001>

12. Mikulska M, Furfaro E, Del Bono V, Gualandi F, Raiola AM, Molinari MP, Gritti P, Sanguinetti M, Posteraro B, Bacigalupo A, Viscoli C. 2012. Galactomannan testing might be useful for early diagnosis of fusariosis. Diagn Microbiol Infect Dis 72:367-9. <https://doi.org/10.1016/j.diagmicrobio.2011.12.009>

13. Inano S, Kimura M, Iida J, Arima N. 2013. Combination therapy of voriconazole and terbinafine for disseminated fusariosis: case report and literature review. J Infect Chemother 19:1173-80. <https://doi.org/10.1007/s10156-013-0594-9>

14. Nakai K, Yoneda K, Imataki O, Kida J, Uemura M, Moriue T, Kubota Y. 2014. Transepidermal growth in disseminated Fusarium infection. J Dermatol 41:770-1. <https://doi.org/10.1111/1346-8138.12555>

15. Rose SR, Vallabhajosyula S, Velez MG, Fedorko DP, VanRaden MJ, Gea-Banacloche JC, Lionakis MS. 2014. The utility of bronchoalveolar lavage beta-D-glucan testing for the diagnosis of invasive fungal infections. J Infect 69:278-83. <https://doi.org/10.1016/j.jinf.2014.04.008>

16. Garcia-Ruiz JC, Olazabal I, Adan Pedroso RM, Lopez-Soria L, Velasco-Benito V, Sanchez-Aparicio JA, Navajas A, Montejo M, Moragues MD. 2015. Disseminated fusariosis and hematologic malignancies, a still devastating association. Report of three new cases. Rev Iberoam Micol 32:190-6. <https://doi.org/10.1016/j.riam.2014.11.003>

17. Hu CH, Curry EJ, Matzkin EG, Todd DJ, Cai AN, Milner DA, Jr., Sparks JA. 2015. Monoarthritis in a 28-Year-Old Man With Juvenile Idiopathic Arthritis. Arthritis Care Res (Hoboken) 67:1328-34. <https://doi.org/10.1002/acr.22597>

18. Koltze A, Rath P, Schoning S, Steinmann J, Wichelhaus TA, Bader P, Bochennek K, Lehrnbecher T. 2015. beta-D-Glucan Screening for Detection of Invasive Fungal Disease in Children Undergoing Allogeneic Hematopoietic Stem Cell Transplantation. J Clin Microbiol 53:2605-10. <https://doi.org/10.1128/JCM.00747-15>

19. Garcia RR, Min Z, Narasimhan S, Bhanot N. 2015. Fusarium brain abscess: case report and literature review. Mycoses 58:22-6. <https://doi.org/10.1111/myc.12271>

20. Ricna D, Lengerova M, Palackova M, Hadrabova M, Kocmanova I, Weinbergerova B, Pavlovsky Z, Volfova P, Bouchnerova J, Mayer J, Racil Z. 2016. Disseminated fusariosis by Fusarium proliferatum in a patient with aplastic anaemia receiving primary posaconazole prophylaxis - case report and review of the literature. Mycoses 59:48-55. <https://doi.org/10.1111/myc.12421>

21. Angebault C, Lanternier F, Dalle F, Schrimpf C, Roupie AL, Dupuis A, Agathine A, Scemla A, Paubelle E, Caillot D, Neven B, Frange P, Suarez F, d'Enfert C, Lortholary O, Bougnoux ME. 2016. Prospective Evaluation of Serum beta-Glucan Testing in Patients With Probable or Proven Fungal Diseases. Open Forum Infect Dis 3:ofw128. <https://doi.org/10.1093/ofid/ofw128>

22. Azoulay E, Guigue N, Darmon M, Mokart D, Lemiale V, Kouatchet A, Mayaux J, Vincent F, Nyunga M, Bruneel F, Rabbat A, Bretagne S, Lebert C, Meert AP, Benoit D, Pene F. 2016. (1, 3)-beta-D-glucan assay for diagnosing invasive fungal infections in critically ill patients with hematological malignancies. Oncotarget 7:21484-95. <https://doi.org/10.18632/oncotarget.7471>

23. Sheela S, Ito S, Strich JR, Manion M, Montemayor-Garcia C, Wang HW, Oetjen KA, West KA, Barrett AJ, Parta M, Gea-Banacloche J, Holland SM, Hourigan CS, Lai C. 2017. Successful salvage chemotherapy and allogeneic transplantation of an acute myeloid leukemia patient with disseminated Fusarium solani infection. Leuk Res Rep 8:4-6. <https://doi.org/10.1016/j.lrr.2017.07.001>

24. Uemura S, Tamura A, Yamamoto N, Saito A, Nakamura S, Fujiwara T, Tahara T, Kozaki A, Kishimoto K, Ishida T, Hasegawa D, Muraosa Y, Kamei K, Kosaka Y. 2018. Successful Combination Therapy of Liposomal Amphotericin B and Caspofungin for Disseminated Fusariosis in a Pediatric Patient With Acute Lymphoblastic Leukemia. Pediatr Infect Dis J 37:e251-e3. <https://doi.org/10.1097/INF.0000000000001941>

25. Yoshida M, Kiyota N, Maruyama K, Kunikata H, Toyokawa M, Hagiwara S, Makimura K, Sato N, Taniuchi S, Nakazawa T. 2018. Endogenous Fusarium Endophthalmitis During Treatment for Acute Myeloid Leukemia, Successfully Treated with 25-Gauge Vitrectomy and Antifungal Medications. Mycopathologia 183:451-7. <https://doi.org/10.1007/s11046-017-0221-x>

26. Dehal N, Quimby D. 2019. Disseminated Fusariosis in a Patient With Acute Myeloid Leukemia: A Case Report. Cureus 11:e5922. <https://doi.org/10.7759/cureus.5922>

27. Konuma T, Takahashi S, Kiyuna T, Misawa Y, Suzuki M, Isobe M, Jimbo K, Mizusawa M, Kato S, Takahashi S, Tojo A. 2019. Fungemia due to Fusarium solani under low-dose liposomal amphotericin B in a patient after cord blood transplantation. J Infect Chemother 25:635-8. <https://doi.org/10.1016/j.jiac.2019.02.020>

28. Nucci M, Barreiros G, Reis H, Paixao M, Akiti T, Nouer SA. 2019. Performance of 1,3-beta-D-glucan in the diagnosis and monitoring of invasive fusariosis. Mycoses 62:570-5. <https://doi.org/10.1111/myc.12918>

29. Szvalb AD, Kontoyiannis DP. 2019. Acute acalculous cholecystitis due to Fusarium species and review of the literature on fungal cholecystitis. Mycoses 62:847-53. <https://doi.org/10.1111/myc.12953>

30. Biddeci G, Dona D, Geranio G, Spadini S, Petris MG, Pillon M, Biffi A, Putti MC. 2020. Systemic Fusariosis: A Rare Complication in Children with Acute Lymphoblastic Leukemia. J Fungi (Basel) 6. <https://doi.org/10.3390/jof6040212>

31. Fernandez-Cruz A, Semiglia MA, Guinea J, Martinez-Jimenez MDC, Escribano P, Kwon M, Rodriguez-Macias G, Chamorro-de-Vega E, Rodriguez-Gonzalez C, Navarro R, Galar A, Sanchez-Carrillo C, Diez-Martin JL, Munoz P. 2020. A retrospective cohort of invasive fusariosis in the era of antimould prophylaxis. Med Mycol 58:300-9. <https://doi.org/10.1093/mmy/myz060>

32. Fujishita K, Oka S, Kamei K, Tani K, Fujisawa Y, Kitamura W, Machida T, Imai T. 2020. A Disseminated Fusarium fujikuroi Species Complex Infection Prior to Allogeneic Hematopoietic Stem Cell Transplantation. Acta Med Okayama 74:435-41. <https://doi.org/10.18926/AMO/60805>

33. Hoshino A, Tokoro S, Akashi T, Inoue M, Takagi M, Imai K, Kanegane H, Muraosa Y, Kamei K, Morio T. 2020. Disseminated fusariosis in a child after haploidentical hematopoietic stem cell transplantation. Pediatr Int 62:419-20. <https://doi.org/10.1111/ped.14097>

34. Ning JJ, Li XM, Li SQ. 2021. Disseminated Fusarium bloodstream infection in a child with acute myeloid leukemia: A case report. World J Clin Cases 9:6049-55. <https://doi.org/10.12998/wjcc.v9.i21.6049>

35. Brent G, Abdul-Wahab A, Borman AM, Ferguson L, Ferreras-Antolin L, Ho B, Johnson EM, Mashhoudi Y, van Rijswijk E, Wijesuriya N, Mansoor N. 2022. Disseminated Bisifusarium infection following toxic epidermal necrolysis in a child with B-cell acute lymphoblastic leukemia. Pediatr Dermatol, 10.1111/pde.15179. <https://doi.org/10.1111/pde.15179>

36. Delliere S, Guitard J, Sabou M, Angebault C, Moniot M, Cornu M, Hamane S, Bougnoux ME, Imbert S, Pasquier G, Botterel F, Garcia-Hermoso D, Alanio A. 2022. Detection of circulating DNA for the diagnosis of invasive fusariosis: retrospective analysis of 15 proven cases. Med Mycol 60. <https://doi.org/10.1093/mmy/myac049>

37. Jabr R, Liesman RM, Sethapati VR, Shoemaker DM, Spec A, El Atrouni W. 2022. Disseminated Infection Due to Neocosmospora (Fusarium) falciformis in a Patient with Acute Myelogenous Leukemia. Kans J Med 15:67-9. <https://doi.org/10.17161/kjm.vol15.15921>

38. Risum M, Overgaard UM, Rubek N, Haastrup EK, Hare RK, Helweg-Larsen J. 2022. Disseminated fusariosis with cerebral involvement in a patient with acute myeloid leukemia: Successful outcome with intrathecal -and systemic antifungal treatment. J Infect Chemother 28:1324-8. <https://doi.org/10.1016/j.jiac.2022.04.017>

39. Kao AS, Cramer-Bour C, Kupsky W, Soubani AO. 2023. Endophthalmitis as the initial manifestation of invasive fusariosis in an allogeneic stem cell transplant patient: A case report. Med Mycol Case Rep 40:5-7. <https://doi.org/10.1016/j.mmcr.2023.02.004>

40. Ochiai N, Shimazaki C, Uchida R, Fuchida S, Okano A, Ashihara E, Inaba T, Fujita N, Nakagawa M. 2003. Disseminated infection due to Scedosporium apiospermum in a patient with acute myelogenous leukemia. Leuk Lymphoma 44:369-72. <https://doi.org/10.1080/1042819021000029957>

41. Ohashi R, Kato M, Katsura Y, Takekawa H, Hoshika Y, Sugawara T, Yoshimi K, Togo S, Nagaoka T, Seyama K, Takahashi K, Tsuchiya K, Misawa S, Kikuchi K. 2011. Breakthrough lung Scedosporium prolificans infection with multiple cavity lesions in a patient receiving voriconazole for probable invasive aspergillosis associated with monoclonal gammopathy of undetermined significance (MGUS). Med Mycol J 52:33-8. <https://doi.org/10.3314/jjmm.52.33>

42. Takeuchi M, Yoshida C, Ota Y, Fujiwara Y. 2011. Deep skin infection of Scedosporium apiospermum in a patient with refractory idiopathic thrombocytopenic purpura. Intern Med 50:1339-43. <https://doi.org/10.2169/internalmedicine.50.4890>

43. Yu Z, Hu L, Jiang M, Ning H, Xu C, Li B, Li Y, Lou X, Wang J, Hu J, Chen H. 2013. Dermatic Scedosporium apiospermum infection after autologous bone marrow transplantation. Intern Med 52:689-93. <https://doi.org/10.2169/internalmedicine.52.8532>

44. Nishimori M, Takahashi T, Suzuki E, Kodaka T, Hiramoto N, Itoh K, Tsunemine H, Yarita K, Kamei K, Takegawa H, Takahashi T. 2014. Fatal fungemia with Scedosporium prolificans in a patient with acute myeloid leukemia. Med Mycol J 55:E63-70. <https://doi.org/10.3314/mmj.55.E63>

45. Uno K, Kasahara K, Kutsuna S, Katanami Y, Yamamoto Y, Maeda K, Konishi M, Ogawa T, Yoneda T, Yoshida K, Kimura H, Mikasa K. 2014. Infective endocarditis and meningitis due to Scedosporium prolificans in a renal transplant recipient. J Infect Chemother 20:131-3. <https://doi.org/10.1016/j.jiac.2013.09.006>

46. Inoda S, Sato Y, Arai Y, Obata H, Suzuki J, Kaburaki T, Kamei K. 2015. [Bilateral Endogenous Fungal Subretinal Abscesses due to Scedosporium prolificans: a Case Report]. Nippon Ganka Gakkai Zasshi 119:632-9.

47. Ochi Y, Hiramoto N, Takegawa H, Yonetani N, Doi A, Ichikawa C, Imai Y, Ishikawa T. 2015. Infective endocarditis caused by Scedosporium prolificans infection in a patient with acute myeloid leukemia undergoing induction chemotherapy. Int J Hematol 101:620-5. <https://doi.org/10.1007/s12185-015-1752-x>

48. Tamaki M, Nozaki K, Onishi M, Yamamoto K, Ujiie H, Sugahara H. 2016. Fungal meningitis caused by Lomentospora prolificans after allogeneic hematopoietic stem cell transplantation. Transpl Infect Dis 18:601-5. <https://doi.org/10.1111/tid.12563>

49. Levesque E, Rizk F, Noorah Z, Ait-Ammar N, Cordonnier-Jourdin C, El Anbassi S, Bonnal C, Azoulay D, Merle JC, Botterel F. 2017. Detection of (1,3)-beta-d-Glucan for the Diagnosis of Invasive Fungal Infection in Liver Transplant Recipients. Int J Mol Sci 18. <https://doi.org/10.3390/ijms18040862>

50. Kondo M, Goto H, Yamanaka K. 2018. Case of Scedosporium aurantiacum infection detected in a subcutaneous abscess. Med Mycol Case Rep 20:26-7. <https://doi.org/10.1016/j.mmcr.2018.01.003>

51. Mitomo H, Sakurada A, Matsuda Y, Notsuda H, Watanabe T, Oishi H, Niikawa H, Maeda S, Noda M, Sado T, Amemiya T, Yoshida Y, Kikuchi T, Kamei K, Okada Y. 2018. Endobronchial Topical Amphotericin B Instillation for Pulmonary Chromomycosis After Lung Transplantation: A Case Report. Transplant Proc 50:939-42. <https://doi.org/10.1016/j.transproceed.2017.12.028>

52. Sakata Y, Taga F, Ushigami T, Takeda K, Anzawa K, Nishibu A, Mochizuki T. 2018. A Case of Cutaneous Mycosis Caused by Scedosporium dehoogii on an Immunocompromised Patient. Mycopathologia 183:465-70. <https://doi.org/10.1007/s11046-017-0198-5>

53. Bronnimann D, Garcia-Hermoso D, Dromer F, Lanternier F, French Mycoses Study G, Characterization of the isolates at the N. 2021. Scedosporiosis/lomentosporiosis observational study (SOS): Clinical significance of Scedosporium species identification. Med Mycol 59:486-97. <https://doi.org/10.1093/mmy/myaa086>

54. Suzuki Y, Oishi H, Matsuda Y, Noda M, Kumata S, Hayasaka K, Okada Y. 2021. Pneumonia With Scedosporium apiospermum and Lomentospora prolificans in a Patient After Bilateral Lung Transplantation for Pulmonary Hypertension: A Case Report. Transplant Proc 53:1375-8. <https://doi.org/10.1016/j.transproceed.2021.02.002>

55. Bourlond B, Cipriano A, Regamey J, Papadimitriou-Olivgeris M, Kamani C, Seidel D, Lamoth F, Muller O, Yerly P. 2022. Case report: Disseminated Scedosporium apiospermum infection with invasive right atrial mass in a heart transplant patient. Front Cardiovasc Med 9:1045353. <https://doi.org/10.3389/fcvm.2022.1045353>

56. Gao S, Ma X, Kang Y, Zhang Z, Zhang Y, Zhou W, Shen H. 2022. Brain abscess caused by Scedosporium boydii in a systemic lupus erythematosus patient: A case report and literature review. Indian J Med Microbiol 40:611-5. <https://doi.org/10.1016/j.ijmmb.2022.06.010>

57. Garnham K, Halliday CL, Rai NJ, Jayawardena M, Hasan T, Kok J, Nayyar V, Gottlieb DJ, Gilroy NM, Chen SC. 2022. Introducing 1,3-beta-d-glucan for screening and diagnosis of invasive fungal diseases in Australian high-risk haematology patients: is there a clinical benefit? Intern Med J 52:426-35. <https://doi.org/10.1111/imj.15046>

58. Saini V, Shah A, Jaber T, Como J, Min Z, Bhanot N. 2022. Scedosporium apiospermum mediastinitis in an orthotopic heart transplant recipient. IJID Reg 5:117-20. <https://doi.org/10.1016/j.ijregi.2022.09.011>
